# Inflammatory signaling on cytochrome P450-mediated drug metabolism in hepatocytes

**DOI:** 10.3389/fphar.2022.1043836

**Published:** 2022-10-24

**Authors:** Xiaokang Wang, Jiaoyu Rao, Zhiyi Tan, Tianrong Xun, Jingqian Zhao, Xixiao Yang

**Affiliations:** ^1^ Department of Pharmacy, Shenzhen Longhua District Central Hospital, Shenzhen, China; ^2^ Department of Pharmacy, Shenzhen Hospital, Southern Medical University, Shenzhen, China; ^3^ Guangzhou Customs Technology Center, Guangzhou, China

**Keywords:** cytochrome P450, inflammatory signaling, drug metabolism, hepatocytes, clinical application

## Abstract

Cytochrome P450 (CYP450) enzymes are membrane-bound blood proteins that are vital to drug detoxification, cell metabolism, and homeostasis. CYP450s belonging to CYP families 1–3 are responsible for nearly 80% of oxidative metabolism and complete elimination of approximately 50% of all common clinical drugs in humans liver hepatocytes. CYP450s can affect the body’s response to drugs by altering the reaction, safety, bioavailability, and toxicity. They can also regulate metabolic organs and the body’s local action sites to produce drug resistance through altered drug metabolism. Genetic polymorphisms in the CYP gene alone do not explain ethnic and individual differences in drug efficacy in the context of complex diseases. The purpose of this review is to summarize the impact of new inflammatory-response signaling pathways on the activity and expression of CYP drug-metabolizing enzymes. Included is a summary of recent studies that have identified drugs with the potential to regulate drug-metabolizing enzyme activity. Our goal is to inspire the development of clinical drug treatment processes that consider the impact of the inflammatory environment on drug treatment, as well as provide research targets for those studying drug metabolism.

## Introduction

The absorption (A), distribution (D), metabolism (M), and elimination (E) of ADME vary widely between individuals, contributing to a variety of drug-induced and environmental exposure-related toxicities, which control the fate of these compounds *in vivo* (Roberts et al., 2018). Individual responses to drugs and environmental exposures can be explained by changes in ADME processes. Many examples indicate that these differences play an important role in a genetic subpopulations’ susceptibility to specific toxicities. However, the possibility of transient phenotypic transformation resulting from changes in the environment, such as inflammation and disease, is often overlooked (Morgan et al., 2020). It is becoming increasingly apparent that disease and the physiological state of the patient, as well as genetic and environmental factors, have an impact on drug metabolism and disposition. The clinical outcome of a disease can be profoundly affected by the expression and activity of drug-metabolizing enzymes and transporters (Xie 2020).

Cytochrome P450 (CYP450) enzymes are membrane-bound, heme-containing terminal oxidases essential for human drug metabolism (M), cellular metabolism, and homeostasis. Nearly 80% of the oxidative metabolism of clinical drugs and approximately 50% of their elimination are attributed to one or more CYPs belonging to CYP families 1 through 3 (Cacabelos and Martínez-Bouza 2011; Koike et al., 2021; Zhao et al., 2021). Genetic polymorphisms may be responsible for the variation observed among races and between individuals. However, recent studies have shown that environmental factors, such as inflammatory status, chemotherapeutic drug exposure, and gut microbiota, can also provide new insights into the mechanism of individual differences in drug treatment and targets, which can be used to prevent adverse reactions (Aitken et al., 2007; Gong et al., 2018; Gomes et al., 2021). Only a handful of studies have addressed clinical problems resulting from these disease states and a systematic study, including of their effects on hepatocyte metabolism, has not been conducted. This review summarizes and highlights the most recent research on hepatic inflammation and the regulation of expression and activity of CYP enzymes during drug metabolism. Nuclear transcription factors and signaling pathways are also discussed to highlight the inflammatory signaling pathways that control the metabolism and elimination of CYP enzymes in hepatocytes, as well as to provide drug targets for future research.

Individualized drug therapy is a constant topic in modern clinical pharmacy. To achieve the most effective use of various drugs for the benefit of the patient, individualized treatment protocols are necessary (Josefsson et al., 2019). The liver is adversely affected by proinflammatory cytokines such as interleukin-1β (IL-1β), IL-6, and tumor necrosis factor (TNF-α); however, the impact of their adverse effect on CYP450 enzyme activity has not been systematically reported. (Renton et al., 2005; Dickmann et al., 2012). IL-6 has specifically been shown to regulate various CYPs, including CYP3A4 (Dickmann, et al., 2012). Clinical inflammatory markers, such as C-reactive protein (CRP), are associated with a variety of “negative acute phase reactions” that involve most drug metabolism enzymes and transporters, which supports the documented negative effects of inflammation on drug metabolism (Harvey and Morgan 2014; Wang et al., 2021). The mechanism leading to synergistic downregulation of drug-metabolizing enzymes and transporters (DMET) was studied in primary human hepatocytes (PHH) treated with IL-6 and selective signal transduction inhibitors (Schröder et al., 2013). MAPK and PI3K were more important for DMET regulation than JAK/STAT signaling, according to reverse-phase phosphoproteomic analysis. The RXR protein was also found to be downregulated, but not its transcript, in response to IL-6. Numerous miRNAs were also found to be significantly elevated in cholestatic liver, including miR-130b, which has previously been shown to suppress the activities of various cytochromes P450 enzymes during inflammation (Rieger et al., 2015). It appears that miR-130b also directly targets RXRα.

**FIGURE 1:**
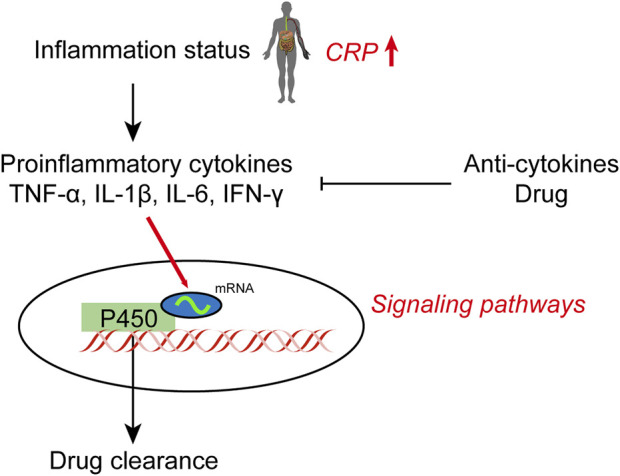
Effect of inflammation on hepatic cytochrome P450 expression and drug clearance.

These results suggest that downregulation of DMET genes in hepatocytes during inflammation involves MAPK and PI3K signaling activation by IL-6, as well as inactivated or downregulated RXRα/nuclear receptor complexes. Lipopolysaccharide (LPS) and various cytokines, including IL-1β, IL-2, IL-6, IFN-γ and TNF-α, also play a role in metabolism. Treatment with TNF-α results in significantly lower expression of CYP2C8 and CYP2C76 mRNA in hepatocytes. IL-1β treatment significantly reduces the expression of CYP1A1, CYP2C8, CYP2C19, and CYP2C76 mRNA in hepatocytes, while it increases the expression of CYP3A5. Cytokines can affect the expression level of CYP450 mRNA in the liver and affect CYP450 expression in humans (Uno et al., 2020). These studies provide deeper insight into the mechanisms controlling CYP450s, specifically their activity, and provide evidence that may allow for the more effective use of clinical drugs (Figure 1).

### Inflammation in hepatocytes

Hepatocytes are protected by Kupffer cells (KCs), endothelial cells, stellate cells, and the perisinusoidal space (Disse) barrier. Pathogen-associated molecular patterns (PAMPs), particularly lipopolysaccharides (LPSs) located in the outer membrane of Gram-positive bacteria, are associated with chronic liver disease, which is largely controlled by factors that contribute to its onset and progression. In chronic alcohol disease, LPS production in the intestine is transferred to the liver, where it combines with hepatocyte death to cause systemic inflammation, aggravating liver disease (He et al., 2021). Around 15% of the liver’s cells are resident macrophages, known as KCs, which play a central role in preventing gut-derived pathogens from spreading throughout the body (Sierro et al., 2017). Around 15% of the liver’s cells are resident macrophages, known as KCs, which play a central role in preventing gut-derived pathogens from spreading throughout the body. (Xu et al., 2022).

**FIGURE 2:**
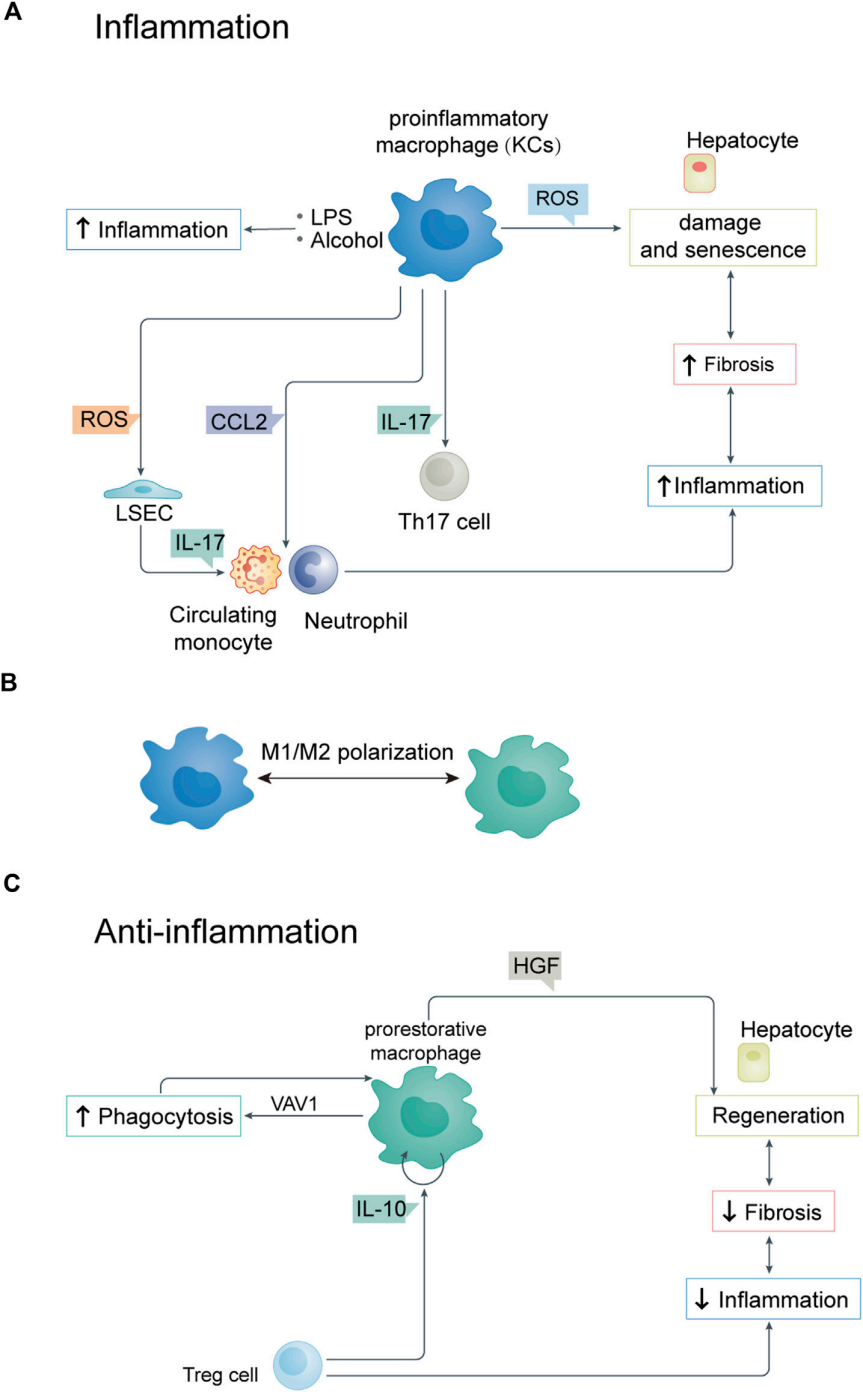
**(A)** Relationship between parenchymal and non-parenchymal cells during inflammation. **(B)** M1/M2 polarization of KCs. **(C)** Relationship between parenchymal and non-parenchymal cells during anti-inflammation. LSEC, liver sinusoidal endothelial cell.

Liver macrophages, such as KCs, are activated by the convergence of alcohol and LPS. Indeed, stimulation of KCs with alcohol and LPSs can increase proinflammatory cytokines, including IL-1β, IL-6, and TNF-α. Hepatocytes, neutrophils, and macrophages can produce reactive oxygen species more rapidly when LPS interacts with alcohol (Figure 2A) (Brenner et al., 2013). By activating NLRP3 inflammasomes, reactive oxygen species (ROS) cause hepatocyte damage and senescence, resulting in ROS production. ROS also play a role in the immune response of liver sinusoidal endothelial cells (LSECs) (Lao et al., 2018). LSECs are special capillary endothelial cells involved in maintaining metabolic and immune homeostasis and function as a structural barrier. They also participate in the liver immune response by regulating leukocyte (monocyte or neutrophil) recruitment and infiltration into tissues. If KCs are damaged by excessive BAs, they produce ROS, inflammation, and apoptotic factors that lead to LSEC dysfunction by negatively affecting viability and cell characteristics. The loss of functional LSEC may aggravate liver disease because it plays a central role in these processes (Shetty et al., 2018).

Macrophage polarization also plays an important role in the inflammation and anti-inflammatory response of hepatocytes (Figure 2B). The regulation of M1/M2 polarization of KCs is closely related to the progression of liver inflammation (Han et al., 2017). By inhibiting necroptosis-S100A9-necroinflammation, M2-like macrophages play a hepatoprotective role in acute and chronic liver failure (Bai X et al., 2021). Studies on cellular immune regulation have shown that phagocytosis is key to resolving any inflammatory reaction. The low-throughput phagocytosis that occurs during this period leads to dead cells being rapidly cleared by hepatocytes.

Macrophages are mainly responsible for clearing aging red blood cells, neutrophils, and effector CD8^+^ T lymphocytes from the liver (Uderhardt et al., 2019). Macrophages are professional phagocytes responsible for clearing dead cells from the liver after injury. Apoptotic cells respond to “eat me” signals through cell surface receptors on macrophages. During phagocytosis, liver macrophages acquire a “recovery-promoting” phenotype, contributing to liver fibrosis remodeling (Ramachandran et al., 2012). In addition to regulatory T cells, other immune cell types produce anti-inflammatory cytokines which control and terminate inflammation. These include Treg cells, which produce IL-13 that triggers macrophages to produce IL-10. IL-10 acts on macrophages in a cell autonomous manner and activates *VAV1*, which in turn activates RAC1. Studies have shown that hepatic RAC1 activation is necessary for phagosome formation and phagocytosis of apoptotic cells, thus maintaining immune homeostasis (Figure 2C) (Pohlmann et al., 2018; Proto et al., 2018).

### The NLRP3 inflammasome: a metabolic sensor for inflammation

Inflammation is believed to regulate drug metabolism and transporters in a significant way (Stanke-Labesque et al., 2020). The activation of the inflammasome contributes significantly to hepatocyte damage, as well as immune cell activation and inflammation amplification. Inflammasome protein complex activation in immune cells triggers an innate immune response (Murphy 2018). Recent studies have shown that NLRP3 inflammation is involved in the regulation of multiple metabolic enzymes (Zhang et al., 2020; Zhao M et al., 2020). Research on NLRP3 inflammatory activation has become another trend, as it regulates multiple metabolic enzymes, as well as the NF-κB signaling pathway.

**FIGURE 3:**
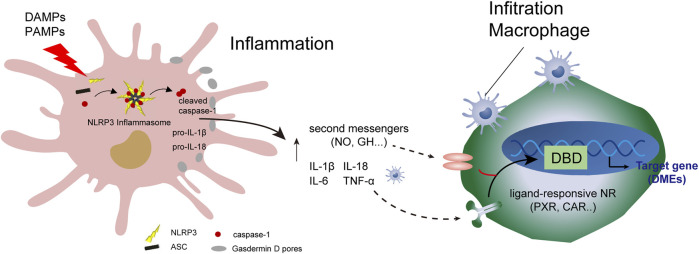
Programmed injury of hepatocytes and regulation of cytochrome 450 enzymes. Crosstalk occurs between parenchymal and non-parenchymal cells during hepatocytic inflammation. DAMPs, damage-associated molecular patterns; DBD, DNA-binding domain.

Controlling inflammation by inhibiting NLRP3 inflammation is key to anti-inflammatory processes (Szabo and Petrasek 2015; Bäck et al., 2019). The endogenous metabolite bile acid can control hepatocyte inflammation and metabolic disorders by inhibiting the NLRP3 inflammasome (Guo et al., 2016). As shown in Figure 3, programmed hepatocyte injury or death (hepatocyte pyroptosis) is a response to PAMPs and danger-associated molecular patterns (DAMPs). The response is usually mediated by inflammasome sensor molecules, such as NLRP3, which is a protein 3 containing the NOD, LRR, and Pyrin domains, as well as ASC receptors and caspase-1.

A protein complex composed of ASC receptors and caspase-1 drives the pro-inflammatory cytokines IL-1β and IL-18, and cleaves Gasdermin D to induce programmed hepatocyte death (Gaul et al., 2021). Programmed hepatocyte injury often induces the recruitment of hepatic macrophages, including KCs. A series of immune responses result in reduced hepatic drug-metabolizing enzyme activity and metabolism, such as lipid metabolism. Analysis of hepatic drug-metabolizing enzymes in disease-state environments and understanding the mechanism of action can help identify the clinical causes of drug metabolism abnormalities and develop biomarkers. These biomarkers can help determine drug-related metabolic enzyme activities, allowing for adjustment of the timing and dosage of drugs, and avoidance of drug-derived adverse reactions.

### Human Hep:KC cocultures: a model of the effect of inflammation on cytochrome P450s in hepatocytes

A coculture model was developed by combining innate immune components to overcome the inherent limitations of hepatocyte single-culture systems such as KCs. Cell cocultures can make up for the defects of monolayer cell culture and are better for observing the interaction between cells and the culture environment. Therefore, cocultures help maintain cellular function and are closer to physiological and pathological models *in vivo* and *in vitro* (Gao et al., 2020). Edward *et al.* believed that cell co-cultures simulate the physiological structure of the human body and provide conditions for cell-cell interaction. The hepatocyte and KC coculture (HKCC) model plays an important role in drug development and metabolic disease research (Rose et al., 2016). Sunman *et al.* demonstrated that a coculture system containing KCs led to IL-2 inhibition of CYP3A activity in hepatocytes for 72 h, while only 48 h of inhibition was observed in a hepatocyte monoculture (Sunman et al., 2004). Similarly, cocultures of parenchymal and non-parenchymal hepatocytes have indicated that KCs affect the upregulation and downregulation of various CYP450 isoforms and transporters (Chen et al., 2011). However, research focused on coculture systems is limited and replicating these observations is challenging. Therefore, establishing a mechanistic research model of the inflammatory state is of great significance for future exploration of CYP450 enzyme activity.

A few studies have characterized established HKCC models. A previously established system was modified to incorporate KCs, which were cultured in empirically optimized collagen micropattern domains supported by mouse 3T3-J2 fibroblasts (Wu et al., 2020). HKCC provides increased stability and allow hepatocytes to maintain their function for several weeks, thus enabling long-term studies. Receptors (i.e., IL-6 and IL-1β) and non-receptors (i.e., IL-2 and IL-23) expressed in hepatocytes have different effects on metabolic enzymes in the Hep:KC coculture system (Nguyen et al., 2015).

### Signaling pathways

#### Nuclear receptors (NRs)

##### Heme oxygenase (HO-1)

Cells are protected from oxidative damage by HO-1, which is activated by substances that cause oxidative stress, including aspirin, statins, and niacin (Abraham et al., 2016). Inflammation, diabetes, liver injury, infectious diseases, and cancer all affect HO-1 levels (Hsu et al., 2006; Abraham, et al., 2016). These diseases are often marked by oxidative stress and induction of HO-1 leads to a reduction of oxidants (heme) and the production of antioxidants, such as bilirubin and carbon monoxide (Connick et al., 2021). Anwar-Mohamed *et al.* found that that arsenite induces HO-1 expression in rat hepatocytes to mediate CYP1A1, CYP1A2, CYP3A23, and CYP3A2 inhibition (Anwar-Mohamed et al., 2012). HO-1 affects cytochrome P450 function in liver through the formation of heteromeric complex CYP1A2·HO-1 (Connick, et al., 2021). Inflammation causes cytokine levels to increase and ROS to build up, resulting in inhibition of HO-1, which depletes its cytoprotective properties. Cytochrome P450 enzymes, specifically CYP2D6, metabolize most drugs used to treat COVID-19 (Fakhouri et al., 2020). HO-1 upregulation has a potential role in the treatment and prevention of cytokine storms. Future research will determine whether inhibition of HO induces CYP2D6 at earlier stages of COVID-19 treatment.

### Peroxisome proliferator activated receptor (PPAR)

PPAR, a nuclear receptor that plays a central role in metabolism, is expressed in tissues with high levels of oxidative stress (Montagner et al., 2016). It has been proposed that inflammation induces CYP4A mRNAs; however, it is unclear if the same mechanism as chemical inducers is used, which involves PPAR activation (Lucarelli et al., 2022). PPARδ agonists can reduce total bile acid content in patients with liver disease. Seladelpar (MBX-8025, a selective PPARδ agonist) has been found to directly activate PPAR, repress liver expression of CYP7A1 (the rate-limiting enzyme for bile acid synthesis), and decrease plasma 7α-hydroxy-4-cholesten-3-one (C4) levels (Jones et al., 2017). PPARα is a ligand-activated transcription factor abundantly expressed in the liver. PPARα activators are reported to prevent acetaminophen-induced hepatotoxicity, which involves regulation of lipid metabolism and inhibition of CYP2E1 activity (Attal et al., 2022). In two previous studies, 2-hydroxyacyl-CoA lyase levels significantly decreased while proteins associated with PPAR signaling, peroxisome proliferation, and omega oxidation, specifically CYP4A10 and CYP4A14, significantly increased in the liver proteome (Mezzar et al., 2017; Khalil et al., 2022).

### Constitutive androstane receptor (CAR)

CARs are members of the nuclear receptor superfamily NR1I3, which are almost exclusively expressed in the liver. The transcription factor *CAR* in human hepatocytes interacts with key signaling pathways involved in drug metabolism and damage prevention in the liver (Tschuor et al., 2016; Bae et al., 2021). CAR regulates numerous genes through its function as a xenobiotic sensor in drug metabolism. By activating *CAR*, the polycyclic aromatic hydrocarbon pyrene induces mouse hepatotoxicity. As a moderate CYP450 inducer, metamizole has a weak inhibitory effect on CYP1A2 and acts as a moderate CYP450 inducer *via* interactions with *CAR* (Bachmann et al., 2021) In a previous study, wild-type mice treated with pyrene had significantly elevated levels of inflammatory cytokines, such as IL-6 and TNF-α, as well as elevated IL-6 serum levels. However, these changes were not observed in *CAR* KO mice (Shi et al., 2022). Therefore, the *CAR* nuclear transcription factor may be involved in the production of inflammatory cytokines, as well as the expression and activity of CYP450 enzymes.

### Aryl hydrocarbon receptor (AhR)

AhR recognizes heterologous and natural compounds that maintain mucosal surface homeostasis, such as tryptophan metabolites, dietary components, and microbial derived factors. As a result of AhR activation, cytochrome P450 1 (CYP1) enzymes are activated to oxidize AhR ligands, which are then metabolically cleared and detoxified (Schiering et al., 2017). CYP1 enzymes have an important feedback role by reducing AhR signals; however, their ability to regulate AhR ligands in hepatocytes is uncertain.

Polycyclic aromatic hydrocarbons and dioxins are persistent organic pollutants that damage the environment and regulate CYP1A1 expression primarily through the transcription factor AhR (Mescher and Haarmann-Stemmann 2018). CYP1A1 activity analysis has been incorporated into modern toxicology concepts and testing guidelines, emphasizing the importance of this enzyme for chemical risk assessment and regulation. The development of CYP1A1 as a molecular target for preventing chemical carcinogenesis is hindered by limitations in the current research process. The main limitation is reduced metabolism of endogenous AhR ligands often observed in various human cancers, which frequently leads to downregulation of CYP1A1 (Al-Dhfyan et al., 2017). Therefore, the endogenous AhR signaling pathway is beneficial for the functional expression of CYP450 and promotes the development of therapeutic drugs.

### Circadian regulation

Diurnal rhythmicity is observed in mice with coumarin hepatotoxicity, with more severe toxicity at ZT2 *versus* ZT14 (ZT, zeitgeber time; ZT2 represents lights on, and ZT14 represents lights off) (Zhao G et al., 2020). The circadian rhythm of drug-metabolizing enzyme expression is considered a key factor affecting circadian metabolism. In general, circadian clock genes regulate the circadian clock by targeting three *cis* elements, specifically the E-box, D-box, and Rev-erbα. The response element or rar-related orphan receptor response element generates and regulates the circadian rhythm of drug-metabolizing enzymes. The clock-controlled nuclear receptors hepatocyte nuclear factor 4α and PPARγ play an important role in circadian enzyme expression. Circadian genes targeted by drugs, such as Rev-erbα, provide an explanation of the genetic variability associated with drug efficacy. BMAL1 and Clock activate the transcription of CYP2A4/5 by directly binding to the E-box C element in the promoter. Knockdown of Clock or Bmal1 downregulates the expression of CYP2A4/5, which are key coumarin-metabolizing enzymes in mice.

Physiological processes, such as inflammation, are coordinated by the biological clock in mammals and recent studies indicate these two pathways interfere with each other. The pro-inflammatory transcription factor κB in the human U2OS cell model was found to regulate the clock by inhibiting the amplitude and duration of the cycle, leading to the phenotype (Shen et al., 2021). Changes in NF-κB also affect the circadian rhythm and motor activity of the suprachiasmatic nucleus depending on the light/dark conditions. The transcriptional activity of circadian E-box transcription factor BMAL1/clock is inhibited by RelA, much like the clock regulator CRY1. The transactivation domain of BMAL1 binds to RelA. NF-κB competes with CRY1 and the coactivator CBP/P300 for BMAL1, thus affecting circadian transcription.

**FIGURE 4:**
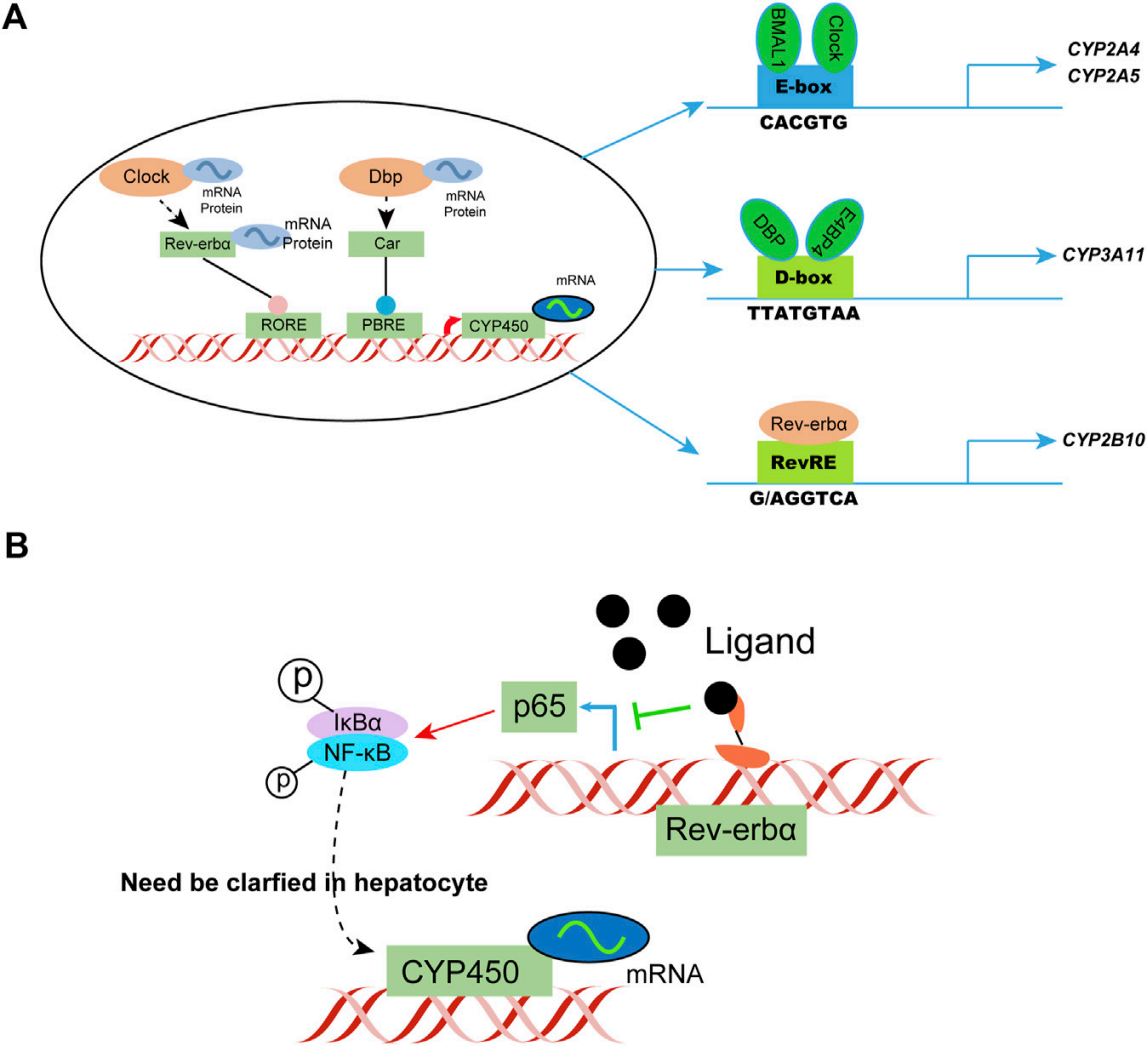
Circadian clock-controlled drug metabolism. **(A)** Molecular mechanisms of circadian clock regulation of CYP450; **(B)** Possible circadian clock regulation of CYP450 in hepatocytes in a state of inflammation. IκBα, I kappa B alpha.

NF-κB assumes a key role in regulating the circadian clock directly and highlights the reciprocal regulation of circadian rhythm and inflammation. Study of the transcriptional effect of NF-κB on BMAL1/Clock in a hepatocyte model, as well as the subsequent effect on CYP450 metabolizing enzyme phenotype, is a new research direction. Additional studies have shown that circadian regulation can alleviate inflammatory responses under physiological and pathological conditions, such as limiting hepatocyte inflammation by daily regulation of oxidative phosphorylation (Bai L et al., 2021). Rev-erbα, a transcriptional repressor regulated by a ligand, is expected to be developed as a therapeutic agent for NASH and other liver diseases (Sato et al., 2020; Griffett et al., 2022). Such studies help predict the circadian pattern of drug metabolism genes in patients with inflammatory conditions (Figure 4).

### Long non-coding RNAs (LncRNAs)

LncRNAs are a special subclass of ncRNAs that make up most of the mammalian genome and are composed of more than 200 nucleotides. They become the main regulators of transcription *via* physical interactions with DNA, other RNAs, and proteins (Long et al., 2017). Numerous lncRNAs are associated with the development of liver disease, as well as inflammation and immune regulation (Chen et al., 2019). Indeed, the chemokine CXCL5 may contribute to the development of NAFLD fibrosis through *MALAT1* lncRNA (Leti et al., 2017). The circadian clock may also regulate lncRNA activity, in addition to the potential effects on circadian biology. Circadian NLRP3 inflammasome activity and steatohepatitis, an NLRP3-related disease, may be regulated by non-oscillatory lncRNAs.

The pluripotency-related transcript (*PLATR*) family is composed of 32 members (*PLATR* 1–32) and has been identified in embryonic stem cells (ESCs), it maintains the gene expression profile of ESCs. In one study, a 4-fold increase in PLATR4 expression was observed in the MMTV-neu NDL breast cancer model, suggesting that PLATR4 may be involved in tumor progression in this model (Diermeier et al., 2016). *PLATR4* is an oscillating lncRNA that is deregulated in murine steatohepatitis. Its oscillation is determined by the diurnal clock component Rev-erbα drive. *PLATR4* acts as a circadian inhibitor of NLRP3 inflammasome activity by inhibiting the transcription and expression of the inflammasome components NLRP3 and ASC. The deletion of *PLATR4* sensitizes mice to experimental steatohepatitis, whereas expression of *PLATR3* improves the pathological conditions. Mechanistically, *PLATR4* prevents NF-κB/Rxrα formation and the κB site, thereby inhibiting the transcriptional activation of NLRP3 and ASC by NF-κB. Therefore, *PLATR4* appears to be an attractive target for the treatment of liver inflammation.

The regulation of inflammation is achieved *via* the main components of the circadian clock, such as Clock, Rev-erbα, and Cry1/2. This explains the circadian rhythm of symptom severity in inflammatory diseases, such as colitis and rheumatoid arthritis, as well as the increased susceptibility to inflammatory-related diseases, such as obesity and NAFLD (Sookoian et al., 2008). *PLATR4* is associated with the NF-κB/NLRP3 inflammasome axis that acts as a circadian clock and an integrating factor of inflammation (Lin et al., 2021). Defining the integrative role of *PLATR4* enhances our understanding of the crosstalk between the circadian clock and inflammation and highlights the complexity of key regulators of inflammation.

### The Ub-proteasome system (UPS)

UPS is an important pathway for selective degradation of intracellular proteins, along with the lysosomal pathway and Ca^2+^-dependent protease pathway. This discovery was recognized with the Nobel Prize in chemistry in 2004. This pathway is composed of ubiquitin, proteasome, and a series of related ubiquitin activating enzymes E1, ubiquitin binding enzyme E2, and ubiquitin ligase E3 (Heo et al., 2022).

In recent years, it has been found that the use of the exogenous NO donor drug NOC18 can also reduce the expression of CYP2B1 protein in primary hepatocytes of rats. However, this degradation process is slower than CYP2B1 reduction induced by IL-1, which induces proteolytic activity (Zheng et al., 2013). Therefore, we speculate that IL-1 may accelerate the protein degradation of CYP2B1 by directly enhancing the activity or expression of the proteasome in the primary hepatocytes of rats. Indeed, Jan *et al.* measured the effect of IL-1 on proteasome activity in such hepatocytes using specific fluorescent polypeptide substrates and found that it improved the chymotrypsin-like activity of the proteasome (Wu et al., 2013). In addition, it can induce the expression of low-molecular-weight polypeptide 2 (LMP2), an important proteolytic subunit of the proteasome, thus revealing the mechanism by which inflammatory cytokines reduce CYP2B1 activity through the proteasome pathway.

### Second messengers

#### Nitric oxide

The *in vivo* synthesis of nitric oxide by nitric oxide synthase (NOS) utilizes the amino acid L-arginine and molecular oxygen. NOSs in the body are divided into three types: neuronal (nNOS), endothelial (eNOS), and inducible synthases (iNOS) (Leo et al., 2021). Infection and inflammation are the main factors that increase the expression of iNOS (Simpson et al., 2022). By reacting with superoxide, peroxynitrite can be produced by NO, which decays into highly toxic hydroxyl radicals. In response to inflammatory stimuli, iNOS is expressed primarily in macrophages, including KCs, and hepatocytes (Navarro et al., 2015). Supernatants from LPS-activated KCs and a specific combination of cytokines and LPS induce iNOS the most in hepatocytes. Glucocorticoids inhibit IL-1 and TNF-induced iNOS induction by hepatocytes, while acute-phase proteins in the liver are differentially regulated by hepatocyte iNOS. In response to iNOS or exposure to eNOS, hepatocyte protein synthesis is reduced and mitochondrial enzymes are partially inhibited (Cho et al., 2018; Wang et al., 2022). The binding of NO to hemoproteins makes it capable of inhibiting the catalytic activity of CYP450 enzymes. Adding NO to liver microsomes from rats reduces CYP450 activity through a partially reversible process (Dey and Cederbaum 2007). The irreversible inhibition is caused by the reaction of NO species with critical amino acid residues. CYP2B1/2 activity is inhibited *in vitro* by NO derived from the NO-generating compound SIN-1, which correlates with the formation of a heme-NO adduct (Yao et al., 2019).

NO derived from induced hepatocytes and KCs has the potential to inhibit hepatic cytochrome P450s and contributes significantly to inflammation-induced reductions in CYP450 catalytic activity. NO may not be necessary for suppressing all CYP450 proteins and mRNAs, as the effects depend on the CYP450 and model of inflammation or infection being studied. It may have greater relevance in endotoxemia, where hepatic iNOS is most induced, *versus* models of remote localized inflammation, where CYP450 is downregulated and iNOS is not induced (Martínez et al., 2018). The contribution of NO to CYP450 activity reduction varies depending on the kinetics of NOS induction relative to *CYP450* mRNA suppression during the inflammatory response phase.

### Nicotinamide and N1-methylnicotinamide (MNAM)

Diabetes mellitus type 2 (T2DM) has become a major global public health concern (Lu et al., 2020). Insulin-resistant states, such as obesity and T2DM, are often associated with chronic, unresolved tissue inflammation (Ying et al., 2020). NASH is closely related to inflammatory changes in the liver and is caused by an endocrine disorder through a mechanism that can affect CYP17A1 (Loria et al., 2009). An endocrine disorder known as polycystic ovary syndrome (PCOS) is associated with hyperandrogenism, insulin resistance, infertility, and ovulation problems. Off-label medicines, such as metformin, are used to treat specific problems caused by PCOS, such as insulin resistance and hyperandrogenism. As a substrate for visfatin and nicotinamide N-methyltransferase (NNMT), nicotinamide produces nicotinamide adenine dinucleotide (NAD) and MNAM, which is an anti-inflammatory, anti-thrombosis, and anti-diabetic agent (Nejabati et al., 2020). In a study that evaluated the effects of MNAM and nicotinamide on metabolic and endocrine abnormalities in a letrozol-induced rat model of PCOS (Nejabati, et al., 2020), the combined effects of MNAM and nicotinamide reversed abnormal estrous cycles and reduced serum levels of testosterone, as well as CYP17A1 gene expression. All of the therapeutic factors improved homeostatic model assessment of insulin resistance scores after treatment, with nicotinamide significantly increasing GLUT4 expression and decreasing visfatin expression. These results indicate that nicotinamide and MNAM have beneficial effects, some of which may be due to the role of AMPK in CYP17A1 expression.

### Growth hormone (GH)

GH is the most important physiological regulator of cytochrome P450 gene expression in rodents. GH receptors belong to a superfamily that includes receptors for a variety of cytokines, such as IL-6. RXRα is an important signaling component recently reported to participate in the sex-dependent effect of GH on CYP3A expression (Li et al., 2015). CYP2C12 accounts for more than 40% of total hepatic cytochrome P450 content in the liver of rats. Octreotide is a potent somatostatin analog and its use increases the level of hepatic ubiquitin-CYP2C12 and significantly inhibits the production of CYP2C12 protein. Therefore, GH can restrict the expression of CYP450 enzyme (Banerjee et al., 2013).

*STAT5a* and *STAT5b* help regulate the activity of the CYP3A10 hydroxylase promoter in response to GH (Subramanian et al., 1998). Cytokines, such as IL-6, affect the ability of GH to control expression of CYP450 by altering the properties of *STAT* heterodimers that bind DNA and activate transcription. The activation of phospholipase A affects the physiological and pathophysiological pathways (cytokines) regulated by CYP450, which is required for the regulation of *CYP2C12* genes by GH. Additional research is needed to understand the regulation of cytochrome P450 gene expression by GH.

### Potential drugs for cytochrome P450

Geniposide (GP) is an iridoid glycoside derived from gardenia fruit that may have profound anti-inflammatory properties against chronic and acute inflammation. Wan *et al.* studied the protective effects and potential mechanism of GP on acetaminophen (APAP) hepatotoxicity. (Li et al., 2016; Yang et al., 2019). GP inhibited CYP2E1 expression and attenuated GSH depletion and MDA accumulation in the liver. Inflammatory cell infiltration was significantly inhibited by GP, as well as the expression and activation of TLR4 and NF-κB, which are receptors for pain signaling. These data suggest that GP downregulates expression of CYP2E1 and the NF-κB signaling pathway to effectively protect hepatocytes from APAP hepatotoxicity (Yang, et al., 2019).

*In vivo* and *in vitro* analyses of curcumin have shown that it inhibits the cytochrome P450 family, including CYP1A2, CYP2C, and CYP3A4. Similarly, experimental data have shown that curcumin significantly reduces HgCl_2_-induced upregulation of CYP450 (Li et al., 2021). These results may reflect the ability of curcumin to act as a chelating agent by directly binding inorganic mercury, which attenuates the biotransformation process of foreign compounds. Curcumin may also inhibit the activation of AhR, which then reduces the expression of CYP1A. CYP450 may therefore play a key role in the protective effects of curcumin in the liver.

Silybin is a flavonoid lignan extracted from the seed coat of *silymarin*, a medicinal plant in the family *Compositae*. It is a CYP3A inhibitor and is extensively used as a hepatoprotective agent in many liver disease therapies. It can inhibit the reduction of CYP3A expression and the activity of conditioned medium (CM) in palmitate-treated KCs (Zhang et al., 2021). It inhibits liver inflammation in mice fed a high-fat diet by increasing *SIRT2* expression and promoting *p65* deacetylation. It also inhibits nuclear factor κB translocation into the nucleus and restores CYP3A transcription affected by CM.

*Tripterygium wilfordii* contains an active compound called triptolide that has antitumor and immunomodulatory properties (Noel et al., 2019). *In vivo* and *in vitro* experiments have indicated that CYP2E1 can mediate oxidative stress and induce hepatotoxicity through a mechanism that promotes oxidative stress, inflammation, and NF-κB (p65) by CYP2E1 (Jiang et al., 2021). Therefore, understanding the CYP2E1 mechanism, its association with NF-κB, and the relationship with the NF-κB (p65) signaling pathway is crucial and meaningful work, which could provide new ideas and effective means for the treatment of hepatotoxic diseases.

Besides natural plant extracts that stimulate CYP450 enzymes, metabolites from human intestinal commensal microbes also participate in hepatocyte metabolic enzyme activity (Zimmermann et al., 2019). Polysaccharide products from intestinal commensal bacteria circulate to the liver along the gut-liver axis and play a regulatory role in drug metabolism in the liver. (Bajaj 2019). *Bacteroides fragilis* polysaccharide A circulates to the liver through hepatic veins and also plays an anti-inflammatory role in the liver. It inhibits proinflammatory factors IL-1β and TNF-α, and upregulates the expression and activity of metabolic enzymes CYP3A4 and CYP2C19, thereby promoting the metabolism of voriconazole and reducing liver toxicity (Wang, et al., 2021).

HepG2 cells treated with rifamycin show elevated PXR transcriptional activity and induced expression of CYP3A4 and P-gp, which are directly responsible for detoxification and are regulated by PXR. Rifamycin also antagonizes TNF-α- and LPS-induced NF-κB activity and inhibits IL-1β-induced synthesis of the inflammatory chemokine IL-8. Although the mutual regulation of PXR and NF-κB by rifamycin has not been directly analyzed, the data indicate that in the absence of PXR, the inhibition of NF-κB by rifamycin is independent of PXR stimulation. Therefore, rifamycin effectively displays anti-inflammatory activity, which is characterized by PXR activation and concomitant induction of CYP3A4 and P-gp *in vitro*, while also inhibiting NF-κB and IL-8 (Rosette et al., 2019).

Certain cytokine-regulated therapeutic proteins, such as gevokizumab, can act by binding to a specific epitope (IL-1β) located close to the receptor interface without completely overtaking the interface. This action may reduce the binding of IL-1β to the IL-1 receptor type I signaling receptor, and the subsequent recruitment of IL-1 helper proteins, primarily through a reduction in the association rate of these interactions. Gevokizumab is thought to be a regulator, rather than a blocker, of IL-1β signaling. Therefore, it may reverse inflammation-mediated inhibition of CYP3A4 and transporters. Fardel *et al.* used primary cultures of human inflammatory hepatocytes, specifically cytokine-treated hepatocytes, to characterize the potential effects of anti-inflammatory drugs on the detoxification pathway of drugs in the liver. Gevokizumab significantly regulates CYP450 and transporter activities (Moreau et al., 2017). The exploration of this *in vitro* mechanism using primary hepatocytes may be the preclinical step needed to predict interactions between gevokizumab and other drugs and may provide an experimental basis for rational drug use in clinics.

The inflammatory cytokine IL-6 activates the Janus kinase (JAK) activation of signal transduction and transcription (STAT) signaling pathway, which inhibits expression of hepatocyte cytochrome P450s and transporters. Therapeutic proteins, such as monoclonal antibodies against IL-6 or its receptors, have been shown to restore the complete detoxification capacity of the liver, leading to inflammatory disease-related drug–drug interactions. Fardel *et al.* found that ruxolitinib, a low-dose JAK1/2 inhibitor currently used for the treatment of myeloproliferative tumors, can inhibit the inhibitory effect of IL-6 on the liver detoxification system (Febvre-James et al., 2018).

IL-6-mediated expression of CYP1A2, CYP2B6, and CYP3A4, as well as transporters NTCP, OATP1B1, and Oct1 mRNA levels, are completely inhibited in primary human hepatocytes and differentiated hepatoma HepaRG cells. These results are in line with the simultaneous recovery of CYP450 and drug transport activities in IL-6-exposed HepaRG cells. Ruxolitinib does not moderate the inhibition of drug detoxification protein mRNA levels by IL-1β. The JAK inhibitor and anti-rheumatoid arthritis complex tofacitinib reverses IL-6-mediated inhibition of CYP450 and transporter mRNA expression. These results indicate that small drugs such as the JAK inhibitor ruxolitinib can specifically counteract the IL-6-mediated inhibition of drug-metabolizing enzymes and drug transporters in cultured human hepatocytes.

Cholesterol 7-hydroxylase (CYP7A1) encodes the rate-limiting step of cholesterol conversion to bile acids in the liver. In one study, acute cholesterol feeding of mice upregulated CYP7A1 by stimulating liver X receptor (Janowski et al., 1996). In that same study, a chronic diet high in cholesterol inhibited the expression of CYP7A1 in the liver of mice by >60% and was associated with 2-fold elevation of cholesterol. However, acute feeding increased liver CYP7A1 expression by > 3-fold. Chronic non-acute cholesterol feeding increased the expression of infectious cytokines TNF-α and IL-1β, which inhibited the expression of CYP7A1 in the liver. It has also been found that chronic feeding of cholesterol activates mitochondrial activated protein kinase (MAP), c-Jun N-terminal kinase (JNK), and extracellular signal-regulated kinase (ERK). *In vitro* studies have indicated that inflammatory cytokines TNF-α and IL-1β have a JNK and ERK pathway-dependent inhibitory effect on CYP7A1. Therefore, chronic feeding of a hypercholesterolemic diet induces inflammatory cytokine activation and liver injury, leading to the inhibition of CYP450 through activation of JNK and ERK signaling pathways (Henkel et al., 2011).

People with chronic kidney disease can also experience changes in drug metabolism in the liver due to uremic toxin accumulation. We have investigated the role of advanced oxidation protein products (AOPPs) in the downregulation of CYP1A2 and CYP3A4. In a rat CKD model, the accumulation of AOPPs in plasma was associated with reduced CYP1A2 and CYP3A4 protein levels. Paracetamol and 6-β-hydroxytestosterone are CYP1A2 and CYP3A4 metabolites and were also found to be significantly reduced in liver microsomes. Hepatocytes treated with AOPPs displayed significant reductions in CYP1A2 and CYP3A4 protein and activity levels.

The effects of AOPPs on p-IKKα/β, p-IκBα, and p-NF-κB on the upregulation of inflammatory cytokine protein levels have been investigated. NF-κB pathway inhibitors BAY-117082 and PDTC abolish the downregulation of AOPPs. These findings suggest that human endogenous metabolite AOPPs stimulate NF-κB by increasing the production of inflammatory cytokines and NF-κB-mediated signaling to downregulate the expression and activity of CYP1A2 and CYP3A4. AOPPs may also alter the clearance of non-renal drugs in CKD patients by affecting metabolic enzymes (Xun et al., 2021).

Screening for potential drug–drug interactions can be accomplished using cytokines released in response to immunomodulators in the blood *ex vivo*. The investigational agonist tilsotolimod stimulates the release of macrophage chemoattractant protein-1 (MCP-1), macrophage inflammatory protein-1α (MIP-1α), and interferon-α2a (INF-α2a) in blood from healthy donors, which stimulates macrophage migration. CYP1A2, CYP2B6, and CYP3A4 enzyme expression and activity are not affected when human hepatocytes are cultured with tilsotolimod. However, cytokines stimulated *via* treatment with tilsotolimod reduce CYP1A2 and CYP2B6 activities. The indirect effects of cytokines induced by tilsotolimod on CYP450 enzymes has been investigated *in vitro*. In one study, the presence of the recombinant human chemokines MCP-1 and MIP-1 did not alter CYP1A2, CYP2B6, CYP2C8, CYP2C9, CYP3A4, or *STAT1* mRNA expression, or CYP1A2, CYP2B6, or CYP3A4/5 enzyme activity, in the Hep:KCs model. However, tilsotolimod increased CYP1A2 and CYP2B6 mRNA levels by 2- and 5-fold, respectively, at concentrations over 2.5 ng/ml, and reduced CYP2B6 enzyme activity by 46%. That study established that while INF-2α is inactivated in human liver cells, it mediates the effects of tilsotolimod on CYP1A2 and CYP2B6 expression (Czerwiński et al., 2019).

### Future research

Different inflammatory models require the characterization of individual CYP450 responses, which makes it clear that not all CYP450s are affected equally. Culturing and stably expanding hepatocytes have been challenging, and as of now, liver organoids can only be generated from biliary epithelial cells and tumoroids formed in primary liver cancer cells (Peng et al., 2018). Simulating organ function with hepatocytes through metabolic function is not yet mature. Sources of inflammatory stimuli should also be considered. In addition to the LPS directed simulating in the hepatocytes, environmental LPS indirect simulating are also responsive to hepatocytes *in vivo*. Markowicz study has shown that smoke contains 10–40 ng LPS per cigarette, with cigarettes containing 20 pg (Markowicz et al., 2014). Dust contaminated with LPS is also inhaled by agricultural workers (Gras et al., 2013). Culturing primary hepatocytes in three-dimensional (3D) environments is currently one of the most popular methods (Hu et al., 2018). A multicellular 3D human primary liver cell culture treated with LPS has been shown to potentiate or inhibit hepatic CYP1A1 induction (Esch et al., 2015), which allows an *in vitro* human disease state model to be established. We have only begun to understand the mechanism of CYP450 regulation. Current research focuses more on stimulating individual hepatocytes, which ignores the interaction between different functional cells in human tissues and organs. There is still much work to be done to clarify the mechanisms that regulate CYP450, including NO, sphingomyelin, and cytokine response elements.

Cytokines and interferons are widely believed to mediate inflammation-induced CYP450 inhibition; other causes of CYP450 downregulation, such as trauma (Cui et al., 2021), acute liver injury (Brown and Merad 2015), and partial hepatectomy (Hu, et al., 2018), need to be studied further. This review only describes the Hep:KCs model, which is commonly used to explore the impact of an inflammatory response on the functional activity of hepatocytes. The application of this technology has great potential and further exploration of the expression of CYP450 through the knockdown or overexpression of the above transcription factors is expected in the future. This will allow for a clearer understanding of the regulatory role of the inflammatory response in the metabolic activity of hepatocytes.

It is important to understand how inflammation affects the activity of CYP450 when using CYP450-substrate drugs to treat patients in a rational and safe manner. Inflammation significantly reduces the activity of CYP450 at the post-transcriptional level. A number of factors contribute to this change, including the lack of production and modification of proteins. By monitoring and studying the role and mechanism of inflammation in CYP450 activity, CYP450-substrate drugs can be correctly used in clinicopathological settings based on theoretical and experimental information.

## Conclusion

Infectious and inflammatory stimuli profoundly affect hepatic CYP450 activity, as well as clinical and toxicological outcomes. While it is possible to induce some CYP450s, most are suppressed in the liver. *In vitro* and *in vivo* studies have continuously reported that traditional Chinese medicine, natural products, and monoclonal antibodies can prevent the inhibition of inflammatory cytokines and upregulate the expression of metabolic enzymes. Metabolites, such as endotoxins LPS and AOPPs, can promote the activity of inflammatory cytokines, downregulate the expression of metabolic enzymes, and regulate the metabolic phenotype (Table 1). Therefore, inflammation models may affect the quality and quantity of CYP450 populations around hepatocytes differently, depending on cytokine profiles and concentrations.

**TABLE 1:** Repots of drug treatment on the expression of cytochrome P450.

No.	Agents	Cytochromes P450	Trends	Related cytokines	Signaling route	Experimental model
1	Geniposide (GP)	CYP 2E1	↑	IL-1β, TNF-α	TLR4/NF-κB	Male C57 mice
2	*B. fragilis* Polysaccharide A	CYP2C19	↑	IL-1β, TNF-α	TLR4/NF-κB	Rats
3	Curcumin	CYP1B1	↓	IL-1β, TNF-α	Nrf2/HO-1	Male Kunming mice
		CYP3A1	↓			
		CYP1A2	↓			
		CYP3A11	↓			
4	Gevokizumab	CYP3A4	↓	IL-1β	(NA)	*In vitro*
5	Triptolide	CYP2E1	↑	IL-6, TNF-α	NF-κB (p65)	Mice
6	Silybin	CYP3A	Restore	(NA)	NAD+/sirtuin 2	(NA)
7	Ruxolitinib	CYP1A2	↑	IL-6	(NA)	HepaRG cells
		CYP2B6	↑			
		CYP3A4	↑			
8	Rifamycin SV	CYP3A4	↑	IL-1β	PXR	HepG2 cells
9	Cholesterol	CYP7A1	↓	TNFα, IL-1β	JNK, ERK	FVB/NJ mice
10	Advanced oxidation protein products (AOPP)	CYP1A2	↓	IL-6, TNF-α	NF-κB	Rats
		CYP3A4	↓			
11	Monocyte chemoattractant protein-1 (MCP-1)	CYP1A2	↓	(NA)	(NA)	Human hepatocytes, *Kupffer* cells
		CYP2B6	↓			
		CYP3A4	↓			
12	INF-α2a (2.0 ng/ml)	CYP1A2	↑	(NA)	STAT1	Human hepatocytes, *Kupffer* cells
		CYP2B6	↓			
13	LncRNA *Platr4*	CYP1/2	↑	IL-1β, IL-6, TNF-α and IL-18	NLRP3/NF-κB signaling	Mice

(NA), Not avaliable.

Hepatocyte membrane receptors, transcription factors, small-molecule RNAs, and other primary and secondary signaling factors may be involved in regulating the expression and activity of CYP450 in the inflammatory environment. Metabolic enzyme activity is usually reduced through the downregulation of CYP450 gene transcription and inhibition of enzyme expression. This makes it possible to determine the impact of the inflammatory environment or abnormal lipid metabolism, such as insulin resistance, on the metabolic enzyme activity of corresponding sensitive drugs. It also provides a reference for the selection of corresponding metabolic enzyme drugs for patients in the clinic. The ultimate goal is to predict the clinical or toxic response of humans and other animals to a given drug or toxin in cases of ongoing infection or inflammation. This review highlights all factors that can affect drug-metabolizing enzymes in the liver that are associated with the inflammatory response signaling pathways.
